# Effect of Dietary Protein Levels on Dynamic Changes and Interactions of Ruminal Microbiota and Metabolites in Yaks on the Qinghai-Tibetan Plateau

**DOI:** 10.3389/fmicb.2021.684340

**Published:** 2021-08-09

**Authors:** XiaoLing Zhang, TianWei Xu, XunGang Wang, YuanYue Geng, Na Zhao, LinYong Hu, HongJin Liu, ShengPing Kang, ShiXiao Xu

**Affiliations:** ^1^Northwest Institute of Plateau Biology, Chinese Academy of Sciences, Xining, China; ^2^University of Chinese Academy of Sciences, Beijing, China

**Keywords:** yak, dietary protein, rumen, microbiota, metabolites

## Abstract

To improve performance and optimize rumen function in yaks (*Bos grunniens*), further knowledge on the appropriate dietary protein levels for ruminal microbiota and the metabolite profiles of yaks in feedlot feeding is necessary. Current understanding of dietary protein requirements, ruminal microbiota, and metabolites is limited. In this study, yaks were fed a low-protein diet (L; 9.64%), middle low-protein diet (ML; 11.25%), middle high-protein diet (MH; 12.48%), or a high-protein diet (H; 13.87%), and the effects of those diets on changes and interactions in ruminal microbiota and metabolites were investigated. Twenty-four female yaks were selected, and the effects on ruminal microbiota and metabolites were investigated using 16s rRNA gene sequencing and gas chromatography time-of-flight/mass spectrometry (GC-TOF/MS). Diets containing different protein levels changed the composition of the rumen bacterial community, the H group significantly reduced the diversity of ruminal microbiota (*p* < 0.05), and the number of shared amplicon sequence variants (ASVs) between the H group and the other three groups was lower, suggesting that the ruminal microbiota community fluctuated more with a high-protein diet. In rumen, *Bacteroidetes*, *Firmicutes*, and *Proteobacteria* were the most abundant bacteria at the phylum level, and *Bacteroidetes* was significantly less abundant in the MH group than in the L and ML groups (*p* < 0.05). *Prevotella_1*, *Rikenellaceae_RC9_gut_group*, and *Christensenellaceae_R-7_group* had the highest abundance at the genus level. *Prevotellaceae* was enriched in the low-protein groups, whereas *Bacteroidales_BS11_gut_group* was enriched in the high-protein groups. Rumen metabolite concentrations and metabolic patterns were altered by dietary protein levels: organic acid metabolites, antioxidant-related metabolites, and some plant-derived metabolites showed variation between the groups. Enrichment analysis revealed that significant changes were concentrated in six pathways, including the citrate cycle (TCA cycle), glyoxylate and dicarboxylate metabolism, and butanoate metabolism. Network analysis showed promotion or restraint relationships between different rumen microbiota and metabolites. Overall, the rumen function was higher in the MH group. This study provides a reference for appropriate dietary protein levels and improves understanding of rumen microbes and metabolites.

## Introduction

The yak (*Bos grunniens*) is a unique species that lives on the Qinghai-Tibetan Plateau (QTP). It is adapted to high-altitude and low-oxygen environments and provides resources such as meat, milk, and fur for local pastoralists (Ding et al., 2014). Female yaks are important for the entire population; they provide enough milk for three meals for the pastoralists and are crucial to the reproduction of the herd. The health and nutritional status of females has a direct impact on young animals. As yaks rely on natural pasture, seasonal nutritional imbalances have a significant impact, especially in winter when pastures are insufficient for their maintenance requirements, causing weight loss and even death (Long et al., 2008). Pastoralists remain heavily impacted by traditional values that affect yak productivity and the ability to resist disasters (such as snowstorms). Therefore, some pastoralists are gradually changing their traditional grazing to indoor feeding or supplements in winter. This has had a positive effect on livestock growth performance, grazing stress, and pastoralist income (Liu P. et al., 2019). However, the lack of nutritional requirements of yak is another major knowledge gap that hinders development of the animal husbandry. Resources for diet and the nutrition (especially the protein and energy content) are extremely important. Ministry of Agriculture of the PRC (2004) was launched due to market demand, allowing the centralized management of beef cattle, but the standard of yaks still needs to be explored.

Rumen function plays a critical role in ruminant production, animal health, and metabolism. The rumen microbiome consists of bacteria, fungi, archaea, and protozoa, which are required to degrade and transform feedstuffs, fermented plant proteins, and polysaccharides. These provide metabolites such as volatile fatty acids (VFAs), amino acids, and saccharides used by organisms to promote growth and enable microbial reproduction (Shabat et al., 2016). This process is equally important for humans because it harnesses the solar energy stored in plant fibers and converts it into milk and meat (Jami et al., 2013). A number of studies have shown that diet is a key factor influencing rumen microbes (Kim et al., 2011; Yeoman et al., 2018). Microbiome studies are becoming increasingly easier, and big data-driven omics research has led to a steep rise in investigations involving two of the most functional omes, the microbiome and metabolome (Misra, 2020). The former includes both the microbiota and its genome, while the latter is a useful method to identify the health status of an organism in nutrition regulation studies, and is widely used in nutritional studies and more frequently used in ruminant studies (Zeisel et al., 2005; Drackley et al., 2006). Therefore, a combination of the microbiota and metabolites could offer important insights. A study of both rumen microbes and metabolites in yak found that the microbiota composition was significantly different in the forge group compared to the concentrate group. This affected the concentration of metabolites and metabolic way, as well as the concentrations of ruminal metabolites participating in protein digestion and absorption (Liu C. et al., 2019). Another study on dairy cow metabolomics found that a high-concentrate diet (70%) increased the concentration of bacterial degradation products, some toxic compounds and 15 amino acids, which changed the rumen metabolic pattern, with more complicated metabolites compared to the low concentrate diet (40%) (Zhang et al., 2017). Although microbiome and metabolome studies have been widely applied in animal nutrition, there is a lack of information on yaks. Thus, improving research on yaks will help us to further understand the rumen environment.

16S rRNA sequencing is an effective method analyzing microbiota with techniques improving steadily with the development of microbiology. Second-generation technology is mature and available to researchers to achieve the research objectives efficiently. Metabolomic analysis methods include nuclear magnetic resonance (NMR), liquid chromatography–mass spectrometry (LC-MS), and gas chromatography–mass spectrometry (GC-MS). Techniques can be selected based on the physical and chemical properties of the sample (Krone et al., 2010; Drexler et al., 2011; Oakman et al., 2011). In recent years, LC-MS and GC-MS techniques have been used extensively. LC-MS is most suited to investigating higher-molecular-weight compounds, but GC-MS is widely used because of its high-throughput capability and high sensitivity (Styczynski et al., 2007; Liu et al., 2010).

In this study, 16S rRNA sequencing and gas chromatograph system coupled with a Pegasus HT time-of-flight mass spectrometer (GC-TOF/MS) were used to explore the effect of dietary protein levels on ruminal microbiota and metabolite profiles in yaks. An association analysis was conducted to identify the relationship between ruminal microbiota and metabolites. It is hypothesized that different protein levels impact the ruminal microbiota and metabolites.

## Methods

### Animals, Diets, and Experimental Design

This study was conducted in January 2019 at the Haibei Demonstration Zone of Plateau Modern Ecological Animal Husbandry Science and Technology, in Qinghai Province, China.

Twenty-four two-year-old healthy female yaks (body weight: 107.54 ± 4.72 kg) were selected from the grazing grassland. Each yak was ear-marked and randomly assigned into four groups. Yaks were fed a low-protein diet (L; 9.64%), middle low-protein diet (ML; 11.25%), middle high-protein diet (MH; 12.48%), or a high-protein diet (H; 13.87%). All yaks were initially fed 1% of their mean body weight. The amount was increased daily until day 15 when the volume of food reached 1.5% of the yak’s body weight. After day 15, the amount of feed was adjusted every 2 weeks based on body weight. The diet was formulated according to the nutrient requirements for dairy heifers (Council, 2007), with varying protein levels and the same metabolizable energy (ME) level. The nutrient composition is provided in Table 1. Yaks were allowed free access to water and fed twice daily at 8:00 and 17:00.

**TABLE 1:** Ingredients and nutrient composition of each diet.

**Ingredients (%)**	**Group**			
	**L**	**ML**	**MH**	**H**
Oat hay	40	40	40	40
Corn	30.3	29.4	28.2	24.6
Wheat bran	21.6	18.6	15.6	15.6
Rapeseed meal	5.1	3.6	1.8	0.6
Corn meal	0.3	2.4	4.8	7.8
Soybean meal	0.3	3.6	7.2	9
Salt	0.6	0.6	0.6	0.6
Premix^3^	0.6	0.6	0.6	0.6
CaHPO4	0.6	0.6	0.6	0.6
CaCO3	0.6	0.6	0.6	0.6
Total	100	100	100	100

**Nutrient composition (%)**				

DM ^1^	82.5	83	85	83.5
CP	9.64	11.25	12.48	13.87
ME mc/kg ^2^	12.04	12.01	11.98	11.95
NDF	38.23	36.70	34.03	33.14
ADF	21.81	19.31	18.10	18.07
Ca	0.67	0.69	0.71	0.73
P	0.52	0.54	0.54	0.59

*DM, dry matter; CP, crude protein; NDF, neutral detergent fiber; ADF, acid detergent fiber.*

*^1^DM is determined based on air-dry basis.*

*^2^ME (metabolizable energy) = total digestible nutrients × 0.04409 × 0.82, according to the National Research Council (Council, 2007).*

*^3^Premix (provided per kilogram of complete diet): vitamin A 200,000 IU; vitamin D3 15,000 IU; vitamin E 1,250 IU; Cu 375 mg; Fe 15,000 mg; Zn 750 mg; Mn 1,000 mg; Se 7.5 mg.*

The crude protein (CP), neutral detergent fiber (NDF), acid detergent fiber (ADF), Ca, and P in each sample were measured in the laboratory, and the ME was calculated. Mixed feed (100 g) was collected and dried in a forced-air oven at 60°C for 48 h, then ground through a 1-mm sieve before analysis. DM and N contents were determined according to AOAC (2007). NDF and ADF contents were analyzed according to Vansoest et al. (1991).

### Rumen Sample Collection and Measurements

At the end of the trial (day 135), rumen fluid samples were collected before the first morning feeding using an oral stomach tube. Rumen samples were strained through four layers of cheesecloth to obtain rumen liquids. Disposable sterile gloves were worn throughout the sample collection process to avoid contamination. Twenty-four samples were collected and placed in 2-ml frozen tubes to avoid cross-contamination. The samples were immediately frozen in liquid nitrogen and stored at −80°C.

### Microbial DNA Extraction, PCR Amplification, Sequencing, and Sequencing Data Processing

Total genome DNA was extracted using the CTAB/SDS method. DNA concentration and purity was monitored on 1% agarose gels. According to the concentration, DNA was diluted to 1 ng/μl using sterile water. The 16S rRNA genes of distinct regions (16S V3–V4) were amplified using specific primers 341F (5′-CCTAYGGGRBGCASCAG-3′) and 806R (5′-GGACTACNNGGGTATCTAAT-3′) with barcode. All PCR reactions were carried out using 15 μl of Phusion^®^ High-Fidelity PCR Master Mix (New England Biolabs), 0.2 μM forward and reverse primers, and approximately 10 ng of template DNA. Thermal cycling consisted of initial denaturation at 98°C for 1 min, followed by 30 cycles of denaturation at 98°C for 10 s, annealing at 50°C for 30 s, elongation at 72°C for 30 s, followed by 72°C for 5 min. The same volume of 1 × loading buffer (containing SYB green) was mixed with the PCR products and electrophoresis was operated on 2% agarose gel for detection. PCR products were mixed in equidensity ratios and then purified with Qiagen Gel Extraction Kit (Qiagen, Germany). Sequencing libraries were generated using TruSeq^®^ DNA PCR-Free Sample Preparation Kit (Illumina, United States) following the manufacturer’s recommendations, and index codes were added. The library quality was assessed on the Qubit@2.0 Fluorometer (Thermo Scientific) and Agilent Bioanalyzer 2100 system. The library was sequenced on an Illumina NovaSeq platform and 250-bp paired-end reads were generated by Novogene Bioinformatics Technology Co., Ltd. (Beijing, China).

The 16S rRNA gene sequences were processed using USEARCH v10.0 (Edgar, 2010) and scripts were written by Liu et al. (2020). Metadata are given in Supplementary Table 1. The quality of the paired-end Illumina reads was checked by FastQC v.0.11.5 (Andrews, 2013) and processed in the following steps by USEARCH: merging paired reads and relabeling of sequencing names, removal of barcodes and primers, filtering of low-quality reads, and finding non-redundancy reads. Unique reads were denoised into amplicon sequence variants (ASVs) by unoise3 in USEARCH (Edgar and Flyvbjerg, 2015). The feature table (ASV table) was generated by VSEARCH (Rognes et al., 2016). The SILVA v123 (Quast et al., 2013) database was used to classified the taxonomy of the representative sequences, and then plastid and non-bacteria were removed.

The alpha diversity and Bray--Curtis distance-based constrained principal coordinate analysis (CPCoA) were accessed using USEARCH v10.0. The alpha diversity boxplot, CPCoA plot, taxonomy barplot, and UpSet plot visualization were performed with ImageGP website^1^. The EdgeR package was used for bacteria gene differential analysis, and group-to-group differential analysis data are displayed as a Manhattan plot.

All the raw sequences after assembling and filtering were submitted to the National Center for Biotechnology Information (NCBI) Sequence Read Archive^2^, under accession number PRJNA706154.

### Preparation of Rumen Sample for GC-TOF/MS and Identification of Compounds

Rumen fluid samples were dried completely in a vacuum concentrator without heating, and then 60 μl of methoxyamination hydrochloride (20 mg/ml in pyridine) was added and incubated for 30 min at 80°C. BSTFA reagent (80 μl, 1% TMCS, v/v) was added to the sample aliquots and incubated for 1.5 h at 70°C. Samples were analyzed by an Agilent 7890 gas chromatograph system coupled with a Pegasus HT time-of-flight mass spectrometer (GC-TOF/MS). The system utilized a DB-5MS capillary column coated with 5% diphenyl cross-linked with 95% dimethylpolysiloxane (30 m × 250 μm inner diameter, 0.25 μm film thickness; J&W Scientific, Folsom, CA, United States). A 1-μl aliquot of analyte was injected in splitless mode. Helium was used as the carrier gas, the front inlet purge flow was 3 ml min^–1^, and the gas flow rate through the column was 1 ml min^–1^. The initial temperature was kept at 50°C for 1 min, then raised to 310°C at a rate of 20°C min^–1^, and then kept at 310°C for 6 min. The injection, transfer line, and ion source temperatures were 280°C, 280°C, and 250°C, respectively. The energy was −70 eV in electron impact mode. The mass spectrometry data were acquired in full-scan mode with m/z of 50–500, at a rate of 12.5 spectra per second after a solvent delay of 4.78 min.

Chroma TOF 4.3X software of LECO Corporation and the LECO-Fiehn Rtx5 database (Kind et al., 2009) were used for raw peaks exacting, data baselines filtering, and calibration of the baseline, peak alignment, deconvolution analysis, peak identification, and integration of the peak area. Both mass spectrum match and retention index match were considered in the metabolite identification.

The resulting data, with the compound name, sample label, and normalized peak area were imported into SIMCA-P software (V14.1, Umetrics AB, Umea, Sweden) for orthogonal projections to latent structures-discriminant analysis (OPLS-DA). Differentially expressed metabolites (DEMs) were identified, combing Variable Importance in Projection (VIP) obtained from OPLS-DA analysis and *t*-test (VIP > 1 and *p* < 0.01). DEMs were further identified and validated by the Bovine Metabolome Database (BMDB^3^) and the Kyoto Encyclopedia of Genes and Genomes (KEGG^4^). To identify the patterns of different metabolites with increasing dietary protein levels, the linear, quadratic, and cubic effects among treatments were evaluated among the different metabolites (Zhang et al., 2020). DEMs were imported into the MetaboAnalyst web server^5^ to view their metabolic pathway distribution and enrichment analysis (Xia et al., 2009).

### Correlations Between Microbial Communities and Rumen Metabolites

The DEMs selected by the previous step and the microbes screened by Edge R were used for interactive analysis in R. Spearman correlations were calculated using the Psych packages. The metabolic correlation network was visualized using the Fruchterman Reingold Algorithm in Gephi 0.9.2 software^6^ (Bastian et al., 2009).

### Statistical Analysis

Statistical analysis was performed using R (v3.6.1) and SPSS 17.0. The linear, quadratic, and cubic effects of treatments were evaluated by the lm function of Estimability package in R, according to Zhang et al. (2020).

### Ethical Consideration

All procedures involving animal care adhered to the guidelines provided by the Institution of Animal Care and the Ethics Committee of the Northwest Institute of Plateau Biology, Chinese Academy of Sciences (NWIPB20160302).

## Results

### Variation in Bacterial Diversity in Different Treatment Groups

**FIGURE 1:**
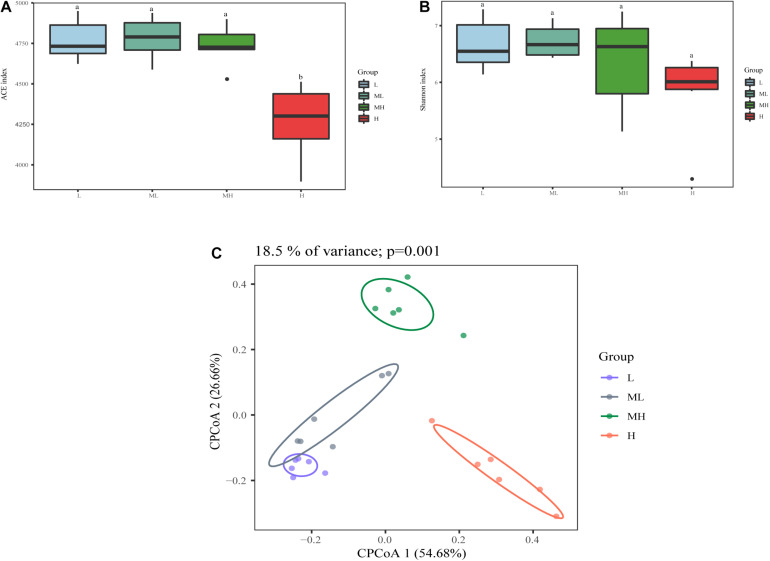
Bacterial diversity analysis of rumen samples between yak fed diets with different protein levels. The alpha diversity between different groups **(A)** ACE index; **(B)** Shannon diversity; beta diversity **(C)** CPCoA plot using Bray–Curtis dissimilarity based on ASVs in different groups.

In total, 2,105,160 raw reads were obtained from the 24 rumen samples, after screening, and 1,500,085 high-quality sequences were obtained (average, 62,503; range, 40,833–71,120 reads per sample). A total of 5600 ASVs were produced based on denoising. The rarefaction curves and richness barplot for the ASVs showed that the quality of the observed species increased with sequencing depth, the H group had the lowest richness of all groups (Supplementary Figure 1). The ACE index was similar in the L, ML, and MH groups, but significantly lower in the H group (*p* < 0.05). The Shannon index had a similar tendency as ACE but was not significant (Figures 1A,B). Analysis of beta diversity with CPCoA (Bray–Curtis distance) showed that rumen microbiota formed four distinct clusters: the L and ML groups separated along the second coordinate axis, while the MH and H groups separated along both coordinate axis with other groups (*p* = 0.001) (Figure 1C). The CPCoA analysis accounted for 18.5% of variance.

### Variation in Bacterial Composition in Different Treatment Groups

**FIGURE 2:**
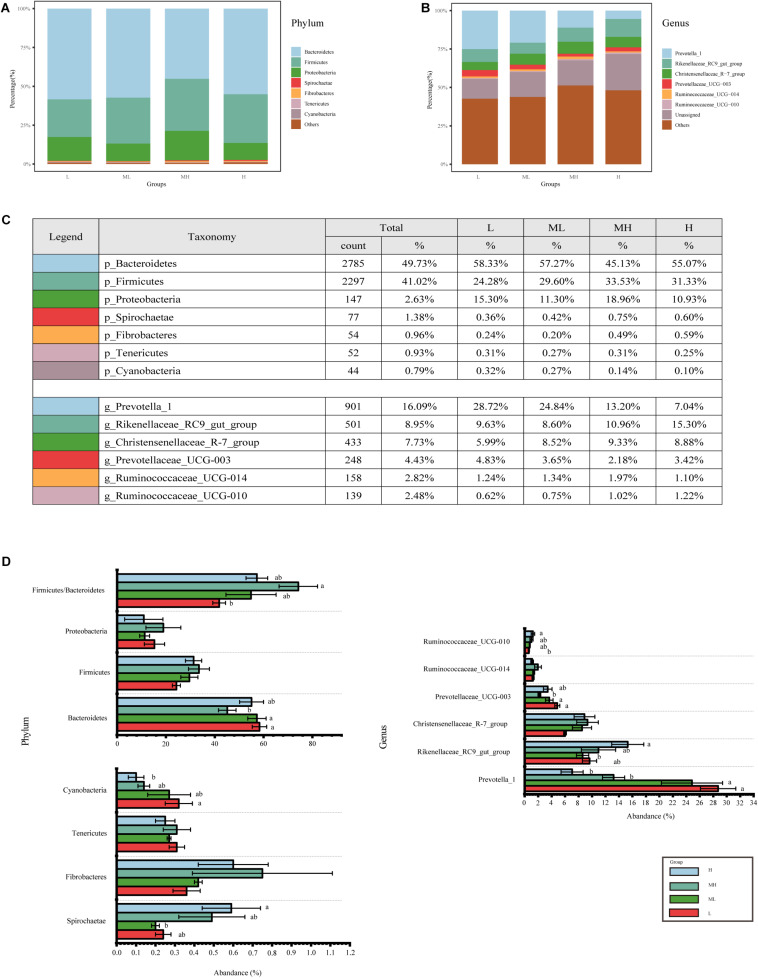
Bacterial composition of rumen samples collected from yaks fed diets with different protein levels. Bacterial composition at the **(A)** phylum and **(B)** genus level, **(C)** further details for the top phylum and genus; **(D)** bacterial genera with significant differences among groups, different letters indicate a significant difference between the groups.

Across all groups, 22 phyla, 44 classes, 73 orders, 102 families, and 183 genera were identified. The relative abundance of dominant taxa at the phylum and genus levels are shown in Figures 2A–C. At the phylum level, the most abundant phyla were *Bacteroidetes* (49.73%), which were significantly less abundance in the MH group than in the L and ML groups (*p* < 0.05), but the H group was not significantly different from the other groups. *Firmicutes* (41.02%) and *Proteobacteria* (2.63%) were enriched in the MH group, but were not significantly differentiated between the other groups (*p* > 0.05). The *Firmicutes/Bacteroidetes* (F/B) ratio in the MH group was higher than other groups and significantly higher than the L group (*p* < 0.05). Less abundant phyla included *Spirochaetae* (1.38%), *Fibrobacteres* (0.96%), *Tenericutes* (0.93%), and *Cyanobacteria* (0.79%), whereas *Fibrobacteres* and *Tenericutes* showed no significant differences between the groups. *Spirochaetae* were higher in the H group and significantly higher than in the ML group. *Cyanobacteria* was lower in the H group compared to other groups, and significantly lower than in the L group. At the genus level, *Prevotella_1* (16.09%), *Rikenellaceae_RC9_gut_group* (8.95%), and *Christensenellaceae_R-7_group* (7.73%) were the predominant genera, *Prevotella_1* was significantly enriched in the L and ML groups compared to the MH and H groups. *Rikenellaceae_RC9_gut_group* was significantly more abundant in the H group than in the ML group. *Christensenellaceae_R-7_group* was highest in the MH group, but not significantly. Other less abundant genera included *Prevotellaceae_UCG-003* (4.43%), *Ruminococcaceae_UCG-014* (2.82%), and *Ruminococcaceae_UCG-010* (2.48%). *Prevotellaceae_UCG-003* was significantly more abundant in the L and ML groups than in the MH group. However, *Ruminococcaceae_UCG-014* showed no significant differences between the groups. *Ruminococcaceae_UCG-010* was significantly higher in the H group compared to the L group (Figure 2D).

### Variation in Bacterial Genus in Different Treatment Groups

**FIGURE 3:**
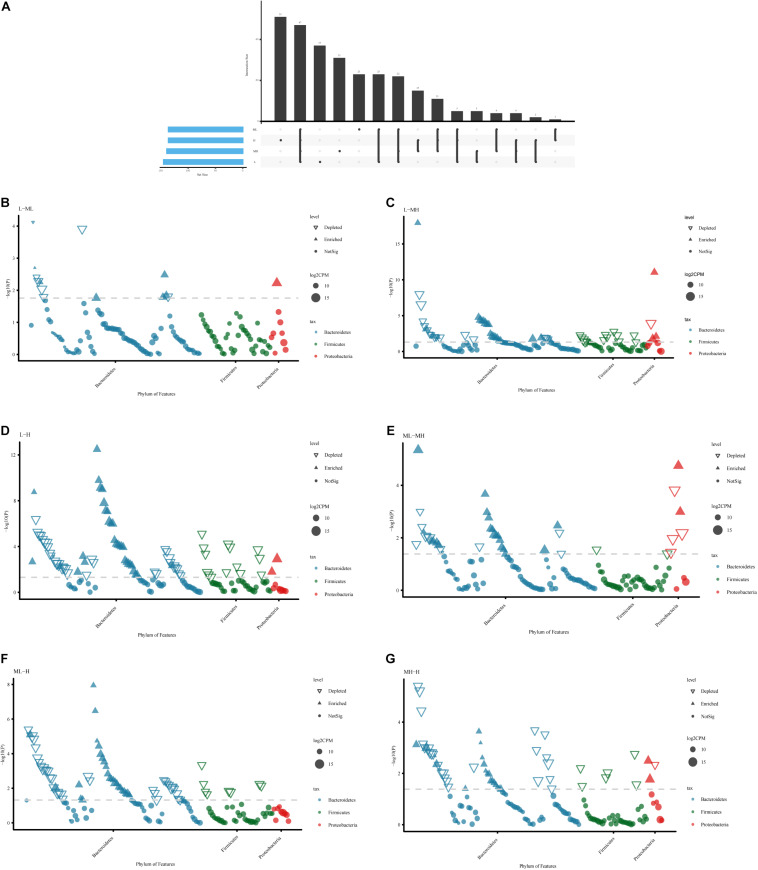
Bacterial variation comparison analysis of rumen samples between yaks fed diets with different protein levels. **(A)** UpSet plot of the ASV count in each group. Comparative analysis between two groups: **(B)** L vs. ML; **(C)** L vs. MH; **(D)** L vs. H; **(E)** ML vs. MH; **(F)** ML vs. H; **(G)** MH vs. H.

In terms of specific bacterial microbiota, 5,600 ASVs were identified; 47 ASVs were shared by all groups, 27 ASVs existed only in the L and ML groups, 15 ASVs were colonized in the MH and H groups, 5 ASVs occurred in just the L and MH groups, and 1 ASV existed only in the ML and H groups. Different groups had different bacterial microbiota. The specific ASVs in each group from large to small are as follows: H (51) > L (37) > MH (31) > ML (23) (Figure 3A). To explore the key microbiota between the groups, the bacterial genera at different protein levels were compared (Figures 3B–G). As protein increased, microbial fluctuations became larger, with changes mainly occurring in *Bacteroidetes*, *Firmicutes*, and *Proteobacteria*. The ASVs belonging to *Bacteroidetes* varied more widely than the other microbes. In low-protein groups, a striking enrichment occurred in most *Prevotellaceae* (family level), whereas there were less *Bacteroidales_BS11_gut_group* and *Rikenellaceae*. Moreover, the *Bacteroidales_UCG-001* and *Christensenellaceae* showed a decreasing trend in the low-protein group (Supplementary Table 2).

### Identification, Quantification, and Statistical Comparison of GC-TOF/MS Metabolites in the Rumen

Supplementary Figure 2 shows the Pearson correlation coefficient between the QC samples. The results confirm the reliable repeatability and precision of the data obtained in this study.

Through GC-MS detection and Chroma TOF software (used for quality control and identification), 202 valid peaks were obtained from the four groups (Supplementary Table 3). These valid peaks were mainly organic acids, lipids and lipid-like molecules, organoheterocyclic compounds, organic oxygen compounds, phenylpropanoids and polyketides, and benzenoids.

**FIGURE 4:**
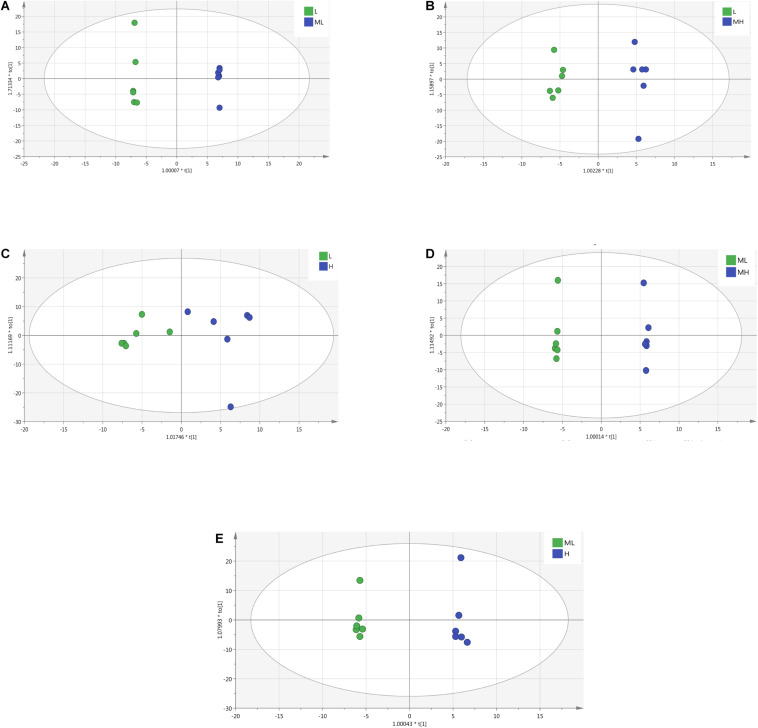
OPLS-DA score plots derived from the GC-TOF/MS metabolite profiles of rumen samples between yak fed diets with different protein levels. **(A)** The L group vs. ML group; **(B)** the L group vs. MH group; **(C)** the L group vs. H group; **(D)** the ML group vs. MH group; **(E)** the ML group vs. H group.

Score plots were created using the OPLS-DA model (Figures 4A–E) to verify the differentiated metabolites between the four groups, and the validation plots (Supplementary Figure 3) are also provided. The corresponding R^2^Y-values of the OPLS-DA models in L vs. ML, L vs. MH, L vs. H, ML vs. MH, and ML vs. H were 0.999, 0.987, 0.849, 0.999, and 0.996, respectively. The MH vs. H the model was not available (Q^2^Y < 0.5). The *R*^2^-values, except MH and H, were all > 0.80, indicating a satisfactory effectiveness of the model. All the samples in the score plots were within the 95% Hotelling T2 ellipse. The score plots revealed clear separation and discrimination between the different protein levels. This indicates that the OPLS-DA model can be used to identify differences between the groups (except MH and H groups).

### Metabolomic Profiles in the Rumen

The combined statistical analysis and VIP value identified 44 differential metabolites (*p* < 0.01 and VIP > 1). Metabolites were classified according to the properties of the compounds, the super class, the mean value, the standard error of the mean (SEM), and the *q* values represented in Table 2. In general, our results show that the main metabolite differences between the four groups were alterations of organic acids and derivatives (12 metabolites), lipids and lipid-like molecules (6 metabolites), organic oxygen compounds (3 metabolites), phenylpropanoids and polyketides (7 metabolites), benzenoids (4 metabolites), organoheterocyclic compounds (2 metabolites), and others (12 metabolites). With increased protein, 3 metabolites (thymol, piceatannol, and hesperitin) cubically increased (*q* < 0.05), biuret significantly decreased, and 12 metabolites (hydroxyurea, 3-hydroxynorvaline, 2-ketobutyric acid, glutaric acid, n, n-dimethylarginine, 4-hydroxybutyrate, dihydrocoumarin, benzoic acid, 4-hydroxybenzoic acid, atrazine-2-hydroxy, l-threose, and n, n-dimethyl-p-phenylenediamine) linearly decreased; the ML group had the highest value and the MH group had the lowest value (*q* < 0.05). Six metabolites [capric acid, 3-phenyllactic acid, hydrocinnamic, 3-(4-hydroxyphenyl) propionic acid, norleucine, and allylmalonic] linearly changed (*q* < 0.05), three metabolites (citramalic acid, maleimide, and 9-fluorenone) quadratically changed, and 4-aminobutyric acid cubically changed (*q* < 0.05) (Table 2). Other responses included a mix of linear, quadratic, and cubic increases and decreases, as shown in Table 2.

**TABLE 2:** Comparisons of ruminal metabolites that significantly changed with yak fed diets containing different protein level ratios.

		**Groups**				**SEM**	***q*-values**			
**Super class**	**Metabolite names**	**L**	**ML**	**MH**	**H**		**Treatment**	**Linear**	**Quadratic**	**Cubic**
Organic acids and derivatives	Hydroxyurea	76,048	148,881	57,813	40,464	11,734.03	< 0.01	<0.001	0.12	0.05
	4-Aminobutyric acid	10,223	29,808	35,768	29,364	3,619.94	0.06	0.36	0.32	0.02
	Isocitric acid	99,404	23,365	23,365	23,365	7,452.25	< 0.001	0.01	< 0.0001	<0.0001
	3-Hydroxynorvaline	528,034	842,436	304,983	189,491	71,817.96	< 0.01	<0.01	0.36	0.01
	Galactonic acid	45,468	63,014	62,004	94,426	6,215.79	0.03	0.12	0.03	0.11
	L-DOPA	10,989	17,795	14,423	19,832	1,157.32	0.02	0.76	0.01	0.18
	2-Ketobutyric acid	105,552	144,046	103,195	98,246	5,463.85	< 0.01	<0.01	0.06	0.18
	4-Acetamidobutyric acid	152,770	194,089	161,726	178,094	5,554.59	0.03	0.20	0.01	0.80
	Citrulline	789,666	1,001,945	797,397	921,408	29,234.05	0.01	0.26	< 0.01	0.79
	3-Hydroxybutyric acid	232,771	315,818	148,691	120,568	24,299.88	0.01	0.01	0.49	0.02
	Glutaric Acid	342,226	476,562	275,466	217,889	32,762.22	0.02	0.01	0.50	0.08
	N,N-dimethylarginine	103,615	175,289	79,589	42,687	17,504.52	0.04	0.01	0.58	0.15
Lipids and lipid-like molecules	Citramalic acid	360,988	507,541	396,597	416,183	18,124.73	0.02	0.03	0.01	0.91
	Thymol	7,920	12,635	18,864	25,635	1,930.52	< 0.01	0.04	0.06	< 0.01
	Capric Acid	146,089	205,068	160,188	154,110	8,461.62	0.05	0.02	0.10	0.90
	Phytanic acid	8,312	21,306	6,921	24,654	2,802.57	0.04	0.61	0.01	0.97
	Arachidic acid	33,435	50,071	33,405	47,824	2,678.01	0.02	0.75	< 0.01	0.91
	4-Hydroxybutyrate	40,263	49,500	24,087	23,951	3,354.78	< 0.01	0.02	0.40	0.01
Organic oxygen compounds	Fucose	288,892	478,845	394,367	363,236	32,282.30	0.22	0.12	0.22	0.48
	Digalacturonic acid	25,798	48,880	36,775	51,166	2,921.60	< 0.01	0.83	< 0.001	0.08
	Tagatose	90,442	166,477	34,926	89,045	16,462.08	0.03	0.18	0.03	0.07
Phenylpropanoids and polyketides	3-Phenyllactic acid	298,914	341,291	304,344	83,639	30,041.52	< 0.01	0.001	0.07	0.25
	Caffeic acid	10,684	22,064	15,670	19,975	1,314.10	< 0.01	0.23	0.001	0.17
	Piceatannol	10,627,937	18,443,030	20,191,325	22,370,834	1425,419	0.01	0.90	0.01	0.02
	Hesperitin	74,725	185,831	201,860	248,675	24,526.29	0.07	0.76	0.09	0.04
	Hydrocinnamic acid	32,692,862	44,812,659	33,903,201	21,915,790	2,634,050.14	0.01	0.002	0.99	0.33
	Dihydrocoumarin	64,748	96,673	43,308	23,238	9,010.95	0.02	0.01	0.70	0.05
	3-(4-Hydroxyphenyl)propionic acid	43,016	70,843	53,067	30,230	4,297.77	< 0.01	<0.001	0.70	0.72
Benzenoids	Pyrogallol	870,419	1,157,283	931,917	1,035,939	33,836.73	< 0.01	0.09	< 0.01	0.80
	Noradrenaline	8,118	21,453	5,912	33,176	3,096.49	0.001	0.08	< 0.001	0.80
	Benzoic acid	494,985	752,846	480,557	307,760	47,527.04	< 0.01	0.0006	0.57	0.15
	4-Hydroxybenzoic acid	12,679	14,534	9,046	7,843	810.81	< 0.01	0.01	0.80	0.01
Organoheterocyclic compounds	Maleimide	170,704	226,584	176,306	182,508	8,172.15	0.05	0.05	0.04	0.68
	Urocanic acid	2,894	5,999	1,941	5,078	457.24	< 0.001	0.54	< 0.001	0.21
Others	Biuret	57,008	40,288	9,128	5,786	6,325.01	< 0.01	0.21	0.31	0.001
	D-Erythronolactone	93,281	116,333	83,983	97,711	3,854.94	0.01	0.12	0.01	0.12
	Mannose	56,689	81,005	37,577	72,988	5,156.19	0.01	0.89	< 0.01	0.09
	Atrazine-2-hydroxy	284,295	389,787	269,739	237,126	20,427.48	0.04	0.01	0.32	0.23
	9-Fluorenone	410,981	538,037	407,150	481,150	20,307.76	0.06	0.32	0.01	0.68
	Norleucine	12,278	20,023	13,683	11,469	1,195.13	0.04	< 0.01	0.20	0.65
	1,4-Cyclohexanedione	163,682	142,916	228,063	88,456	14,010.91	< 0.001	< 0.001	0.81	0.04
	Allylmalonic acid	14,826	20,897	16,422	4,032	2,077.46	0.02	< 0.01	0.38	0.45
	L-Threose	361,272	641,951	339,415	203,554	55,313.76	0.03	< 0.01	0.4541	0.25
	N,N-Dimethyl-p-phenylenediamine	12,025	25,724	11,245	6,745	2,302.25	0.01	< 0.01	0.24	0.22

### Metabolic Pathways of Differential Metabolites

**FIGURE 5:**
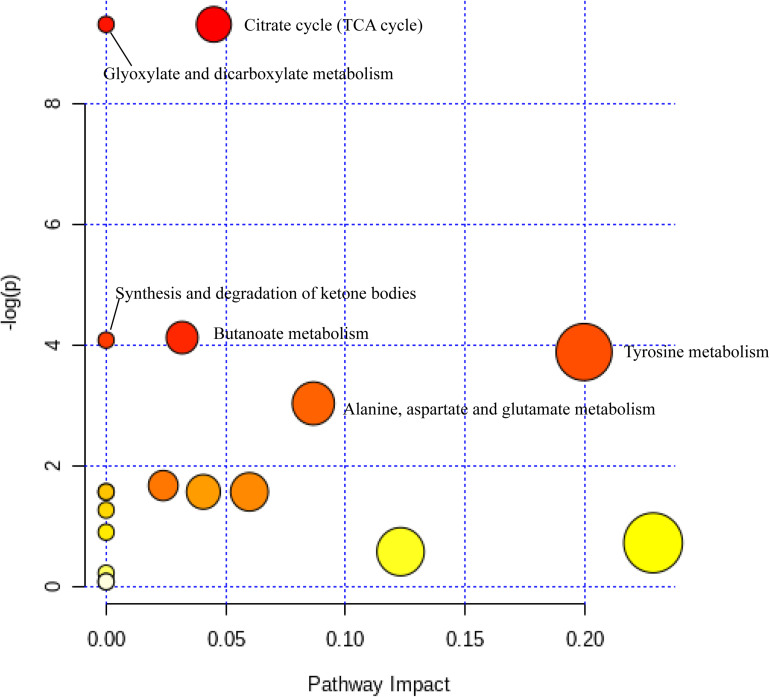
Metabolome view map of the differentially expressed metabolites identified. The deep color shows higher pathway impact values; the larger size demonstrates higher pathway enrichment.

Differential metabolites in rumen samples from the four groups were analyzed using MetaboAnalyst 4.0 see text footnote 5to reveal their association with metabolic pathways. Six pathways, namely, the citrate cycle (TCA cycle), glyoxylate and dicarboxylate metabolism, butanoate metabolism, synthesis and degradation of ketone bodies, tyrosine metabolism, and alanine, aspartate, and glutamate metabolism, were identified as significantly different pathways (*p* < 0.05) (Figure 5). According to the KEGG pathway identification, five metabolites (3-hydroxybutyric acid, 4-aminobutyric acid, isocitric acid, L-DOPA, and noradrenaline) were mapped into these six significant pathways and were therefore identified as key metabolites among the total 44 DEMs.

### Correlations Between the Ruminal Metabolome and Microbiome

**FIGURE 6:**
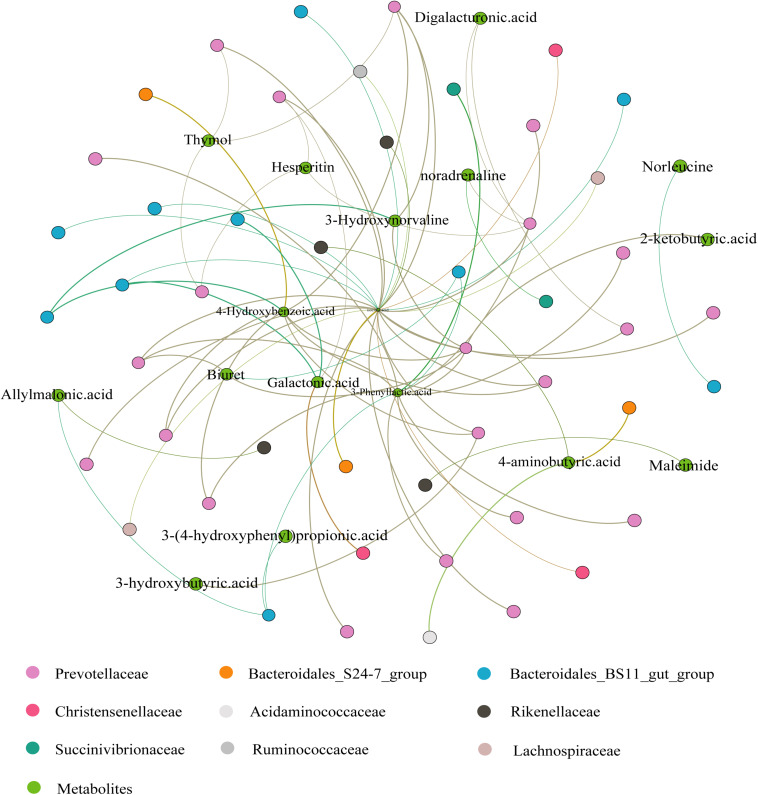
Correlation analysis between the Ruminal Metabolome and Microbiome. The correlations were based on Spearman correlation coefficients (*p* < 0.05). The brown lines to green lines provide the corresponding change from positive to negative correlations.

The correlation analysis of ruminal microbiota and metabolites is provided in Figure 6. Seventeen metabolites (from 44 DEMs) and 48 ASVs (from difference analysis) were screened. The isocitric acid at the center was associated with 25 ASVs (14 positive and 11 negative) and exhibited a positive correlation with most *Prevotellaceae*, but a negative correlation with *Bacteroidales_BS11_gut_group*, *Bacteroidales_S24-7_group*, *Christensenellaceae*, and *Lachnospiraceae*. 3-Phenyllactic acid and 4-Hydroxybenzoic acid showed a positive correlation with *Prevotellaceae*. 3-Phenyllactic acid was negatively associated with *Bacteroidales_BS11_gut_group* but 4-Hydroxybenzoic acid was positively associated. In addition, galactonic acid was positively associated with *Bacteroidales_BS11_gut_group* and *Christensenellaceae*, whereas it was negatively associated with most *Prevotellaceae*. The correlations between other metabolites are shown in Figure 6.

## Discussion

Crude protein is a vital factor in the growth and reproduction of ruminants. Improving the dietary protein level not only promotes yak growth, and the activity of several enzymes, but improves meat composition in Tibetan sheep (Zhang et al., 2014; Wang et al., 2020). In our study, we found that the H group had a negative effect on bacterial richness and alpha diversity, suggesting that a high-quality diet may support lower bacterial diversity, but that lower diversity is not necessarily conducive to a healthy rumen environment (Latham et al., 2018), which is consistent with previous research (Petri et al., 2013). Beta diversity showed a more pronounced specificity cluster, but there was a partial overlap between the L and ML groups, indicating that the two low-protein groups had similar rumen microbiota. Conversely, the MH group and the H group formed distinct rumen flora.

Similar to previous studies (de Menezes et al., 2011; Li et al., 2018; Qiu et al., 2019), *Bacteroidetes*, *Firmicutes*, and *Proteobacteria* were found to be the most abundant bacteria; their higher proportion allowed their members an advantage in the use of nutrients. Among them, *Bacteroidetes* was significantly lower in the MH group than in the L and ML groups, which concurs with previous results where a high nutrient diet decreased the abundance of *Bacteroidetes* (Ley et al., 2006). However, *Firmicutes* and *Proteobacteria* did not vary significantly with protein levels in our study. The F/B ratio is considered a useful indicator of the overall gut bacteria balance and may be involved in maintaining energy homeostasis and suppressing opportunistic pathogens (Guo et al., 2019). For ruminants, the F/B ratio can reflect the host’s ability to absorb and store energy (Turnbaugh et al., 2006; Guo et al., 2008). In this study, the F/B ratio was highest in the MH group; the protein and other nutrients in the diet may have improved the energy absorption capacity of the ruminal bacteria.

At the genus level, *Prevotella_1*, *Rikenellaceae_RC9_gut_group*, and *Christensenellaceae_R-7_group* were the top three species. *Prevotellaceae_UCG-003*, *Ruminococcaceae_UCG-014*, and *Ruminococcaceae_UCG-010* were also detected. *Prevotella* is known as the most abundant bacterial genus in the rumen (Stevenson and Weimer, 2007; Dan et al., 2016) and can produce large amounts of SCFAs by metabolizing dietary fiber in plant cell walls and promote host absorption of monosaccharides by interacting with complex dietary polysaccharides (Ellekilde et al., 2014; Ramayo-Caldas et al., 2016). In the present study, *Prevotella_1* was significantly enriched in the low-protein groups, which could be the result of the higher fiber in the diets, promoting *Prevotella* to efficiently use hemicelluloses to provide more energy for the yaks (Osborne and Dehority, 1989). The *Rikenellaceae_RC9_gut_group* belongs to *Rikenellaceae*, whose members are producers of acetic and propionic acid short-chain fatty acids and ensure that the rumen contains a high percentage of propionic acid (Su et al., 2014). An increase in *Rikenellaceae_RC9_gut_group* in the MH and H groups confirms its important role in protein fermentation (Yang et al., 2020). The *Christensenellaceae_R-7_group* was highest in the MH group. Previous research showed that this genus is more abundant in an alfalfa group than a rice straw group, which indicates a broad link between *Christensenellaceae* and health with improved digestive function (Liu et al., 2016). *Ruminococcaceae_UCG-014* and *Ruminococcaceae_UCG-010* are known to degrade cellulose and protein (FitzGerald et al., 2015), which may explain their enrichment in the high-protein groups.

To further investigate the bacterial characterization in each group, we conducted a differential analysis and discovered that there were more bacterial microbiota present in the H group than in any other group. With this high level of protein, the rumen bacteria were different and less consistent than in the other groups. We also found that in the low-protein groups, most of the *Prevotellaceae* were enriched, while some *Bacteroidales_BS11_gut_group* were depleted. *Prevotellaceae* are responsible for metabolizing dietary fiber from plant cell walls, producing succinic acids for host absorption, and enhancing utilization of the diet (Purushe et al., 2010). *Prevotellaceae* are more abundant in a forage diet (Liu C. et al., 2019). Our study supports this finding. *Bacteroidales_BS11_gut_group* plays an important role in the fermentation of active hemicellulose monomeric sugars and the production of short-chain fatty acids (Solden et al., 2017). We speculated that high-protein diets have an effect on this process, which is consistent with previous research (Liu C. et al., 2019). Additionally, *Rikenellaceae* have the ability to ferment proteins, explaining their higher abundance in the high-protein groups.

The OPLS-DA analysis showed that dietary protein significantly altered the ruminal metabolite composition, suggesting that rumen metabolism is closely related to nutrition. The trends or regression lines between the different groups showed that organic acids were the most significantly affected metabolites in the present study. Lipids, phenolic acids, and aromatic compounds were also detected. In the intestine, proteins are degraded into amino acids, which are then degraded by the microbial flora into a series of downstream products that enter the liver. Amino acids that are not absorbed by the intestine can be fermented by the bacteria to produce organic acids, which are thought to be the intermediate products in the fermentation of sugars by bacteria (Zhao, 2013). The large variation in organic acids in the present study indicates that organic acid metabolism is highly sensitive to nutrient changes. Phenolic acids, such as 3-phenyllactic acid and 3-(4-hydroxyphenyl) propionic acid, are important antioxidants in the host and changes in their metabolic levels imply the changes in the bacteria and the host’s antioxidant capacity (Zhao, 2013). Saleem et al. (2012) reported a decreasing trend in hydrocinnamic acid in high-concentrate grain diets, which is similar to our results. Studies have verified that phytanic acid is derived from chlorophyll and ruminal microorganisms can form phytanic acid by hydrogenation and oxidation of the intermediate product, dihydrophytol. This allows for the slow accumulation of phytanic acid in the body of ruminants, which, as detected in our study, should be attributed to degrade cellulose bacteria (Hansen, 1966; Dawson et al., 1974).

Another metabolite, L-DOPA, is produced by plants and affects the secretion of growth hormones in steers (Kasuya et al., 2017). In the rumen, tyrosine hydroxylase may catalyze the conversion of tyrosine to L-DOPA, allowing the bacteria to use the nitrogen in L-DOPA as a nutrient source in the rumen. L-DOPA increases when the concentration of easily degradable components in the feed is reduced, which may explain the increase on the H diet (Chikagwa-Malunga et al., 2009). However, our knowledge of ruminant metabolites is far less than that of other creatures (Foroutan et al., 2020), and some DEMs detected in our study have not been reported and will require further research.

In terms of further pathway enrichment, the TCA cycle had the largest pathway impact. Isocitric acid and citramalic acid have been established as key players in this pathway, and the two metabolites were significantly decreased on the high-protein diet, appearing that they may be rapidly produced and utilized in this group, promoting the TCA cycle as well as energy conversion (Zhang et al., 2020). Furthermore, isocitric acid also participates in glyoxylate and dicarboxylate metabolism. This pathway is closely associated with the TCA cycle (Lin et al., 2018). A high-protein diet can therefore promote the conversion and absorption of energy in yaks. In addition, the synthesis and degradation of ketone bodies is one of the metabolic pathways associated with the degradation of fatty acids (Zhou et al., 2020). Other pathways related to amino acid metabolism were significantly enriched in this metabolic pathway, indicating that varying dietary protein has a considerable impact on the metabolism of amino acids.

In ruminants, the TCA cycle is central to cellular energy metabolism and chemical synthesis and synthesizes and converts glucose, amino acids, and fatty acids, *inter alia* (Zhu, 2015). In the present study, we observed that isocitric acid (an intermediate product of the TCA cycle) was positively correlated with *Prevotellaceae*. Some members of *Prevotellaceae* can improve feed utilization and produce VFAs to provide more energy, which has a positive effect on the TCA cycle. Therefore, it makes sense that *Prevotellaceae* were enriched in the L group and were correlated with isocitric acid. *Bacteroidales_BS11_gut_group* belongs to *Bacteroidales*, an important specialized anaerobe component of the rumen microbiota that converts pyruvate to acetyl-CoA (Hwang et al., 2017), a key intermediate metabolite of the TCA cycle. This family was negatively correlated with isocitric acid, probably because there is a depletion relationship between the TCA cycle and acetyl-CoA. Galactonic acid (the main component of pectin) was positively associated with *Christensenellaceae* in this study, suggesting that *Christensenellaceae* may be useful in degrading dietary polysaccharides. Moreover, 3-phenyllactic acid has antimicrobial properties and is related to phenylalanine metabolism, and phenylalanine can be catalyzed by phenylalanine hydroxylase to produce tyrosine (Yang et al., 2018; Trost et al., 2020). Our results suggest that *Prevotellaceae* and *Bacteroidales* exert positive and negative effects on the tyrosine metabolic pathway, respectively. A bioactive ingredient in some plants, 4-hydroxybenzoic acid was found to have anti-inflammatory effects in animal trials and human intestinal research (Abbas et al., 2010; Khan et al., 2016). In our study, we also detected these bioactive ingredients, which may be contained in some components of the diet and metabolized in the yak. Unfortunately, their relationships with bacteria require further investigation.

Throughout this study, there were intricate relationships between yak rumen microbes and their metabolites, and both were influenced by dietary protein levels. A previous study indicated that approximately 55–60% of rumen fluid metabolites are associated with the microbiota (Saleem et al., 2013). Other studies have also demonstrated a utilization or productive link between the composition of rumen bacteria and the rumen metabolome (Liu C. et al., 2019; Wang et al., 2019). The varying changes and relationships within the yak rumen in this study revealed important features under the impact of dietary protein level.

In summary, this study combined the microbiome and metabolomics to analyze the correlation between differential bacteria and differential metabolites in the rumen of yaks, and these associations and variations were directly related to dietary protein concentrations. This information enhances our understanding of yak ruminal bacteria and metabolites and provides more information for the development of this field and the protein level requirements within yak diets. In addition, the causes and mechanisms driving the interactions between rumen bacteria and rumen metabolism are worth further investigation.

## Data Availability Statement

The datasets presented in this study can be found in online repositories. The names of the repository/repositories and accession number(s) can be found below: https://www.ncbi.nlm.nih.gov/bioproject/PRJNA706154, accession: PRJNA706154.

## Ethics Statement

The animal study was reviewed and approved by Institution of Animal Care and the Ethics Committee of the Northwest Institute of Plateau Biology, Chinese Academy of Sciences (NWIPB20160302).

## Author Contributions

SX, TX, and XZ: Conception and experiment design. TX, XZ, XW, and YG: experiment conduction. XZ and XW: statistical analysis. SK: resources. XZ: writing – original draft preparation. SX, TX, XW, NZ, LH, and LH: writing – review and editing. All authors have read and agreed to the published version of the manuscript.

## Conflict of Interest

The authors declare that the research was conducted in the absence of any commercial or financial relationships that could be construed as a potential conflict of interest.

## Publisher’s Note

All claims expressed in this article are solely those of the authors and do not necessarily represent those of their affiliated organizations, or those of the publisher, the editors and the reviewers. Any product that may be evaluated in this article, or claim that may be made by its manufacturer, is not guaranteed or endorsed by the publisher.

## Footnotes

1
http://www.ehbio.com/ImageGP/

2
https://www.ncbi.nlm.nih.gov/

3
https://bovinedb.ca/

4
https://www.kegg.jp/

5
https://www.metaboanalyst.ca/

6
https://gephi.org/
