# Supplementary material for: Effect of Dietary Protein Levels on Dynamic Changes and Interactions of Ruminal Microbiota and Metabolites in Yaks on the Qinghai-Tibetan Plateau
Source: Front Microbiol. 2021 Aug 9;12:684340. doi: 10.3389/fmicb.2021.684340 (PMC8381366; doi:10.3389/fmicb.2021.684340)
Supplement: Supplementary file 1 [file Presentation_1.PPTX]

## Slide 1
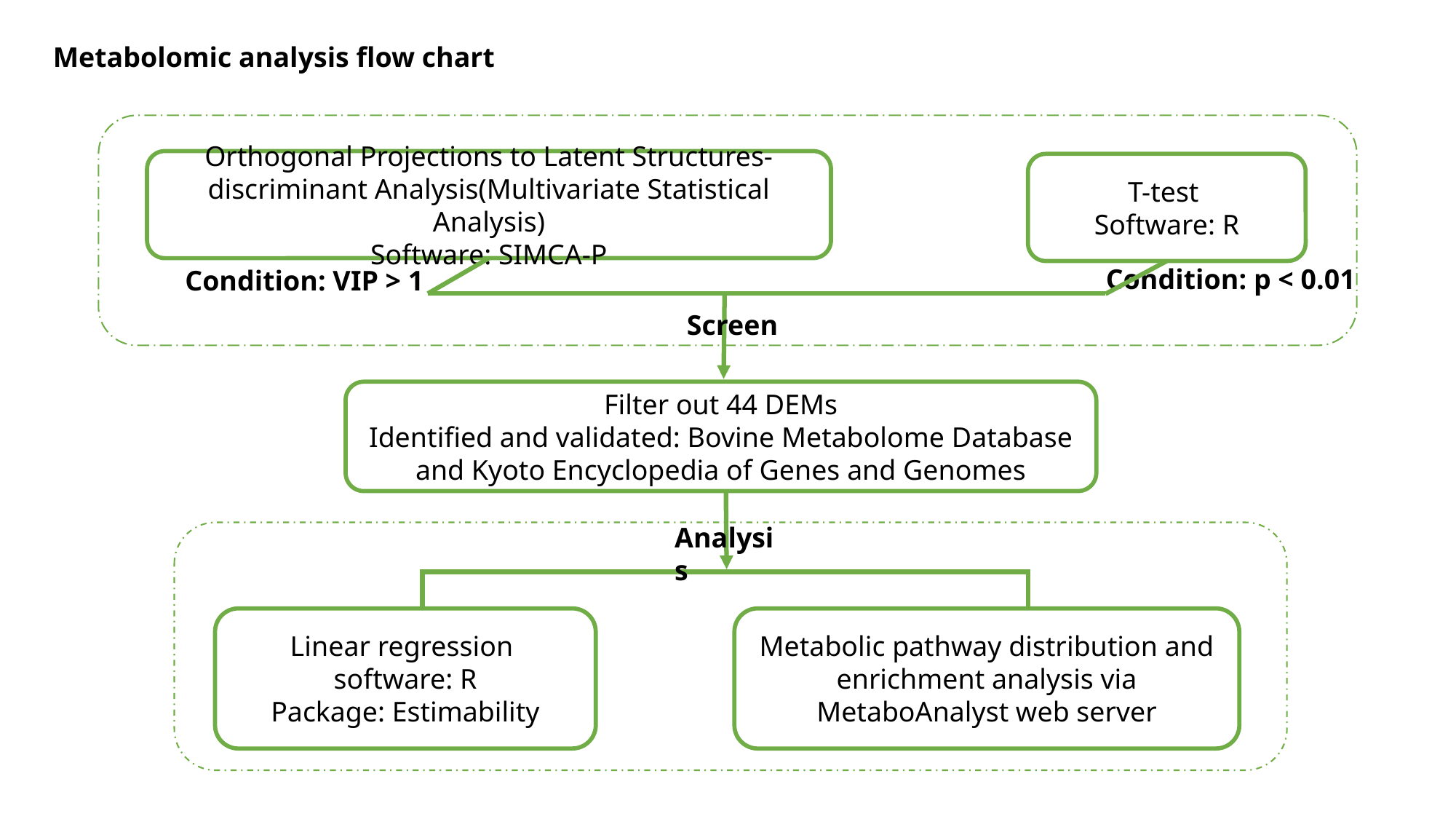

Metabolomic analysis flow chart
Orthogonal Projections to Latent Structures-discriminant Analysis(Multivariate Statistical Analysis)
Software: SIMCA-P
T-test
Software: R
Condition: p < 0.01
Condition: VIP > 1
Screen
Filter out 44 DEMs
Identified and validated: Bovine Metabolome Database and Kyoto Encyclopedia of Genes and Genomes
Analysis
Linear regression
software: R
Package: Estimability
Metabolic pathway distribution and enrichment analysis via
MetaboAnalyst web server
