# Supplementary material for: Effect of Dietary Protein Levels on Dynamic Changes and Interactions of Ruminal Microbiota and Metabolites in Yaks on the Qinghai-Tibetan Plateau
Source: Front Microbiol. 2021 Aug 9;12:684340. doi: 10.3389/fmicb.2021.684340 (PMC8381366; doi:10.3389/fmicb.2021.684340)
Supplement: Supplementary file 2 [file Data_Sheet_1.docx]

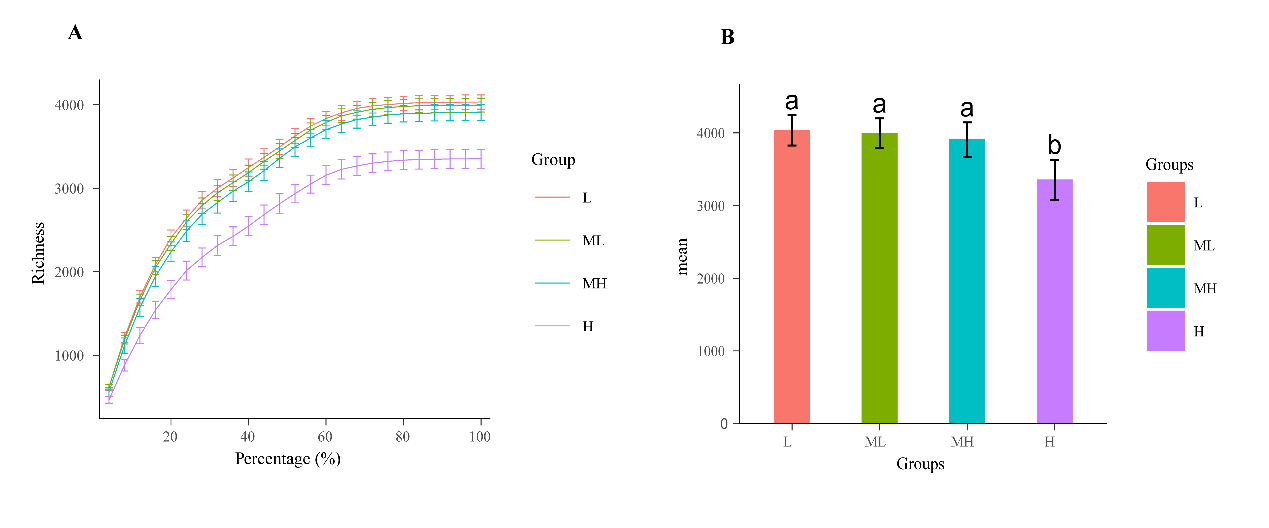


**Figure S1 |** Bacterial rarefaction curves (A) and richness barplot (B) of rumen samples between yak fed diets with different protein level ratios.


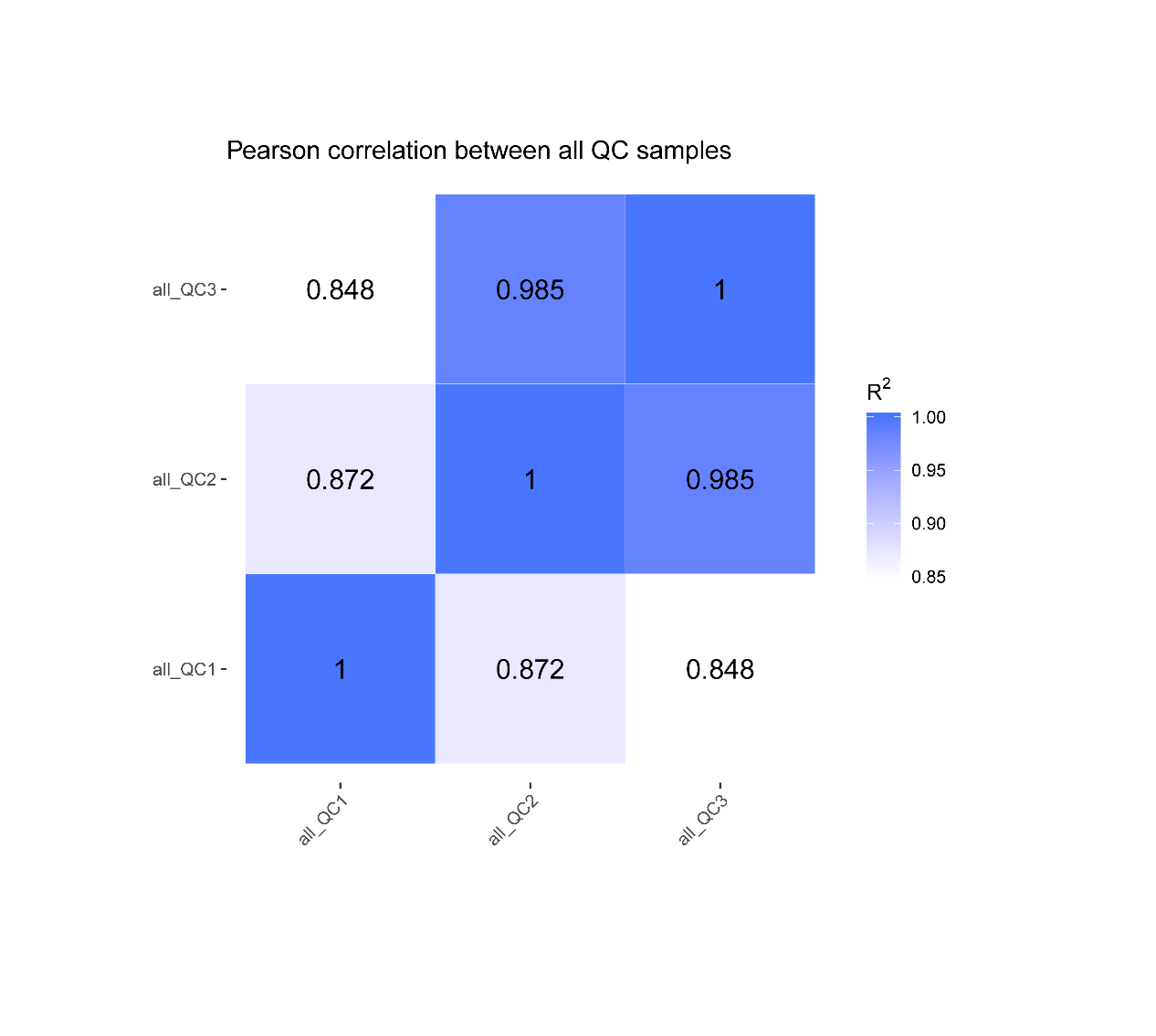


**Figure S2 |** Pearson correlation coefficient between the QC samples. The higher the correlation of the QC sample (R^2^ is closer to 1), the better the stability of the whole detection process and the higher the data quality.


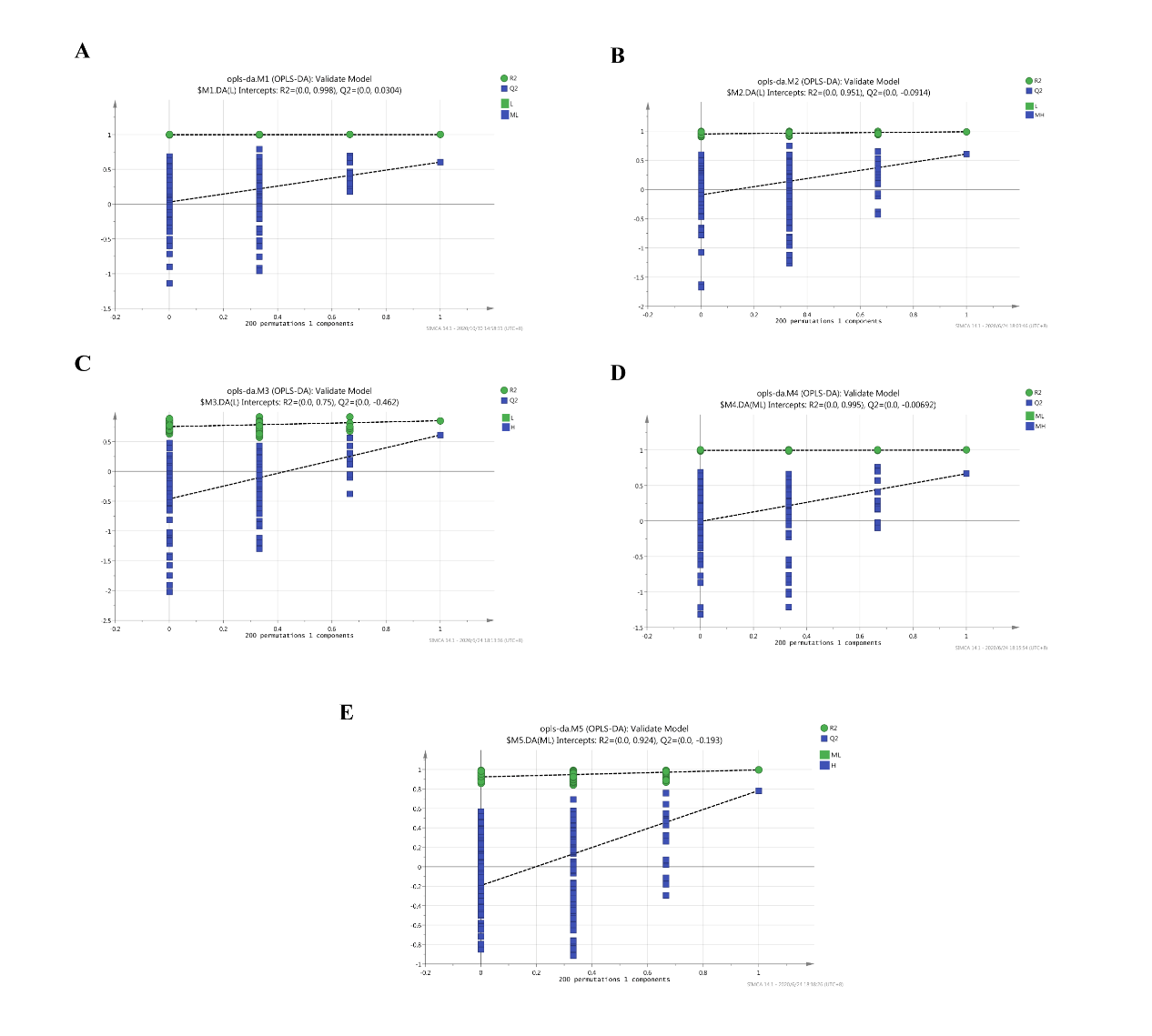


**Figure S3 |** Corresponding validation plots derived from the GC-TOF/MS metabolite profiles of rumen samples between yak fed diets with different protein level ratios. Corresponding validation plots (respectively) for: (A) the L group vs. ML group; (B) the L group vs. MH group; (C) the L group vs. H group; (D) the ML group vs. MH group; (E) the ML group vs. H group. GC-TOF/MS, gas chromatography time-of-ﬂight/mass spectrometry; R2, the interpretability of this model; Q2, the predictability of this model.
